# Examining Mental Health Differences Between Transgender, Gender Nonconforming, and Cisgender Young People in British Columbia

**DOI:** 10.3389/fpsyt.2021.720681

**Published:** 2021-09-29

**Authors:** Rachal Pattison, Joseph H. Puyat, Allison Giesbrecht, Marco Zenone, Steve Mathias, Skye Barbic

**Affiliations:** ^1^Department of Occupational Science and Occupational Therapy, University of British Columbia, Vancouver, BC, Canada; ^2^Centre for Health Evaluation and Outcome Sciences, Providence Health Care Research Institute, Vancouver, BC, Canada; ^3^The University of British Columbia, School of Population and Public Health, Vancouver, BC, Canada; ^4^Foundry, Providence Health Care, Vancouver, BC, Canada

**Keywords:** transgender (GLBT) issues, mental health, gender, integrated services, youth

## Abstract

Foundry is an integrated service network delivering services to young people across British Columbia, Canada. To better understand the needs of transgender and gender nonconforming young people accessing Foundry—this study compares rates of mental health distress between transgender and gender nonconforming young people and cisgender young people accessing services and examines the extent to which race may have amplified the association between transgender and gender nonconforming identity and mental health distress. We analyzed the difference using a two-sample *t*-test. We used stratified simple linear regression to test the association of race with transgender and gender nonconforming identity and mental health distress. Participants were recruited from a network of community health centers in British Columbia, Canada. The quantitative sample (*n* = 727) had a mean age of 21 years (SD = 2), 48% were non-white, 51% were white, and 77% were from Metro Vancouver. Compared to cisgender young people, transgender and gender nonconforming young people reported significantly higher levels of mental health distress. Transgender and gender nonconforming youth were more distressed than cisgender youth across both race strata but non-white transgender and gender nonconforming young people were not more distressed than white transgender and gender nonconforming young people. The findings from this study emphasize the need for increased education and understanding of transgender and gender nonconforming concepts and health concerns as well as on promoting intersectoral collaboration of social services organizations beyond simply health care.

## Introduction

In Canada, people aged 15–24 years have the highest rates of mental health concerns than any other age group and nearly 20% of young people experience a mental disorder compared to 9.8% of those aged 25–64 years (1). In addition to this, young people experience many barriers to accessing health services and with one in four Canadian young people experiencing a mental health difficulty each year, it is crucial that we develop a strong understanding of how to identify young people in distress and the role that the social environment plays in mental health inequities.

To date, much of the research on gender and mental health outcomes has focused on the differences between men and women. A report by the World Health Organization (WHO) titled, Women's Mental Health: An Evidence-Based Review (2) details evidence suggesting that women are twice as likely as men to receive a diagnosis of depression while men are more likely to receive a diagnosis of alcohol dependence. This report also found that women have higher prevalence rates than men in comorbidity of three or more disorders (e.g., depression, anxiety, posttraumatic stress disorder, and agoraphobia, etc.). With this recognition of gender differences in mental health, the WHO further stipulates that individuals who do not fit into the socially constructed categories for gender (i.e., the gender binary of man/woman) face stigma, discrimination, and social exclusion, all of which have a negative impact on health outcomes (3).

This is particularly pertinent to young people who are transgender (identifying with a gender different from the sex assigned at birth) and/or gender nonconforming (TGNC) and whose gender expression may not conform to conventional notions of masculinity or femininity. The difficulties faced by TGNC young people are exacerbated by societal biases, stigma, and conditions favoring cisgender identities (4). The World Professional Association for Transgender Health's (WPATH) Standards of Care emphasizes that “the expression of gender characteristics, including identities, that are not stereotypically associated with one's assigned sex at birth is a common and culturally diverse phenomenon” (5) and encourages the inclusion of a range of gender identities in medical and research approaches. With this in mind, it is vital that we begin to intersectionally (6) understand the impact of gender modality (7) on mental health, so that our Canadian models of youth mental health services delivery be inclusive and responsive to a wide range of needs (8).

There is growing recognition of the importance of integrated health and mental health services for young people in Canada and one such integrated youth service model is called Foundry (foundrybc.ca) (9, 10). The Foundry service model stipulates that health, mental health, and social services are provided all in one location, thus easing the difficulties associated with accessing multiple services at multiple sites (11). In serving young people from ages 12–24 years, Foundry strives to reach individuals as early as possible to better support them in their health and wellness journey. As Foundry works toward expanding across many more communities throughout British Columbia (BC), understanding the mental health outcomes of TGNC youth who access Foundry is critical to the development of more tailored services, accessible to all young people where, and when, they need it.

This study is informed by an intersectional framework that considers “how multiple social identities at the individual level of experience (i.e., micro level) intersect with multiple-level social inequalities at the macro structural level” (12, 13). In keeping with an intersectional approach, this study examines the effect of gender identity on mental health within the context of race. The variable of race was chosen because of evidence that shows being exposed to social advantage and disadvantage (as in adverse experiences like victimization, harassment, discrimination, and/or bullying for example) is implicated in overall mental health and wellbeing (14). This study tested whether TGNC young people accessing integrated mental health services experienced a higher burden of mental health distress compared to cisgender peers. The study's secondary objective was to determine to what extent race amplifies the association between TGNC identity and mental health distress?

## Materials and Methods

### Data Source and Collection

The dataset for this study was pulled from an existing study funded by the Canadian Institute of Health Research, that looked at the mental health and recovery needs of youth accessing Foundry services using the Strategy for Patient-Oriented Research protocol (15). Briefly, it purposively sampled youth from a variety of both urban and rural settings from Foundry locations across BC. The study received ethics approval from the University of British Columbia Research Ethics Board.

### Variables

Gender was explored as the primary independent variable. The dataset defined gender categorically with the following levels: “male,” “female,” “non-binary,” “Two Spirit,” “trans female,” “trans male,” “not sure/questioning,” “prefer not to answer,” and “I don't identify with any of these options/other.” For our study, the cisgender group was characterized by those youth who indicated either “male” or “female” on the primary dataset's gender item. The TGNC group was characterized by those youth who indicated any of the following categories: “non-binary,” “Two Spirit,” “trans female,” “trans male,” “not sure/questioning,” and “other.” The individuals who selected “prefer not to answer” were left out of the analyses to respect their desire to not define their gender; these youth made up 0.005% (4/727) of the entire sample.

The dependent variable was mental health distress, which was captured using the Kessler Psychological Distress Scale (K10). The K10 is a 10-item patient-reported outcome measure designed to capture non-specific psychological (mental health) distress. Each item has a four-point response scale, with a total score that ranges from 0 to 40 (or 10 to 50 depending on how it is scored), with 0 (or 10) indicating no mental health distress and 40 (or 50) indicating the highest mental health distress. Of note, the dataset used in this study scored the K10 from 10 to 50.

Race was selected as an additional variable for study of the intersectional effects of gender and race on mental health (16). In order to maintain adequate numbers for the TGNC category, the data was grouped into two strata: white and non-white groups. The white group was made up of individuals who responded to this item with “White/Caucasian” and the non-white category were those that responded to this item with any other option or with multiple options. Age was also included as a variable of interest because within the wider youth mental health literature, there is some support for the notion that age may be associated with mental health distress, and younger age might infer higher mental health distress (17, 18).

### Data Analysis Strategy

The data was summarized descriptively by examining frequencies, proportions, means, and standard deviations for the two gender identity categories (cisgender and TGNC). A two-sample *t*-test was used to analyze descriptive statistical differences between the two groups. A two-sided significance level of 0.05 was used as means of taking a conservative approach to determining differences between the groups and being open to a difference in either direction. A two-sample *t*-test was selected because it is an appropriate method for comparing two independent groups on a continuous or numeric variable (19), in this case, mental health distress/K10. Also examined was the internal consistency of the K10 item responses in the sample using Cronbach's alpha (20). Internal consistency is a measure of reliability of a set of scale items (i.e., each of the 10 items on the K10) and is an important way to determine if each of these scale items is measuring the same underlying concept (21), in this case, psychological or mental health distress.

Next, a stratified linear regression analysis was used, which examined the primary association of interest (gender identity and mental health distress) stratified by race. Stratification was deemed to be an easier method to interpret, compared to interaction models or more complex hierarchical models, for example. Simple linear regression is typically used when examining the relationship between two numerical variables (22). However, it can also be used to examine differences between groups, for example when looking at the relationship between one categorical variable and one numerical variable (23). Regression was used to adjust for age within this stratified analysis.

For the race variable, a linear regression analysis (both unadjusted and age adjusted models) was conducted within each race stratum: white and non-white. Since the dataset used for this study was not obtained *via* a stratified random sampling method, the data was stratified at the time of analysis. Forty-eight percent (48%) (*n* = 352) of the youth in this sample indicated one or more racial identities other than white, inclusive of Indigenous youth (First Nations, Métis, and Inuit). This is higher than the wider provincial census numbers that show ~36% of British Columbians report being either a visible minority or Indigenous (First Nations, Métis, and Inuit) but similar to proportions seen in Metro Vancouver. The majority of youth who participated in this study (77%) were from the Metro Vancouver area, where demographic reports show 51% of people in Vancouver and metro area report being a visible minority or Indigenous (First Nations, Métis, and Inuit) (24). The sample's strata were considered proportionate to the population from which it was drawn.

## Results

### Demographics

See Table 1 for the characteristics of the study sample. The overall sample had a mean age of 21 years (interquartile range/IQR = 4, standard deviation/SD = 2). The cisgender group comprised 86% (*n* = 627) of the overall sample and the TGNC group made up 13% (*n* = 96) of the sample. Additionally, 0.6% (*n* = 4) of participants reported that they would prefer not to answer the gender identity item. Forty-eight (48%) selected the non-white option(s) for the race item, which were one or more of the following: “First Nations/Métis/Inuit,” “South Asian,” “Black/African,” “Caribbean,” “Hispanic/Latino,” “Middle Eastern/North African,” and “You do not have an option that applies to me/Other.” Other race categories that were specified by participants: “Filipino,” “Vietnam,” “Asian,” “Chinese,” and “Sephardic Jewish.” Over two-thirds of the sample (69%, *n* = 501) had completed at least high school (some high school and completed high school) and 77% of respondents were recruited from Foundry centers in Metro Vancouver, including downtown Vancouver and North Vancouver.

**Table 1:** Characteristics of study sample.

**Age**		***N*** **= 727**
	Mean	21
	Median	21
	Age range	16–25
	IQR**	19, 23
**Gender Identity**		***n*** **(%)**
	Cisgender	627 (86)
	TGNC*	96 (13)
	Prefer not to answer	4 (0.6)
**Race**		
	White	374 (51)
	Non-white	352 (48)
	NA	1 (0.1)
**Non-white subgroups**		***n*** **(%)**
	Indigenous/First Nations/ Métis	116 (16)
	Mixed ethnicity	108 (15)
	South Asian	32 (4)
	Black/African	21 (3)
	Hispanic/Latinx	18 (3)
	Middle Eastern/North African	22 (3)
	Other	23 (3)
	Caribbean	12 (2)
**Level of education**		
	At least high school	501 (69)
	More than high school	222 (31)
	NA	4 (0.6)
**Foundry center**		
	Vancouver Granville	448 (62)
	Vancouver North Shore	108 (15)
	Kelowna	64 (9)
	Prince George	52 (7)
	Victoria	55 (7)
	Campbell River	NA
	Never accessed Foundry	0

* *TGNC = transgender and/or nonconforming*,

** *IQR = Interquartile range*.

**Figure 1:**
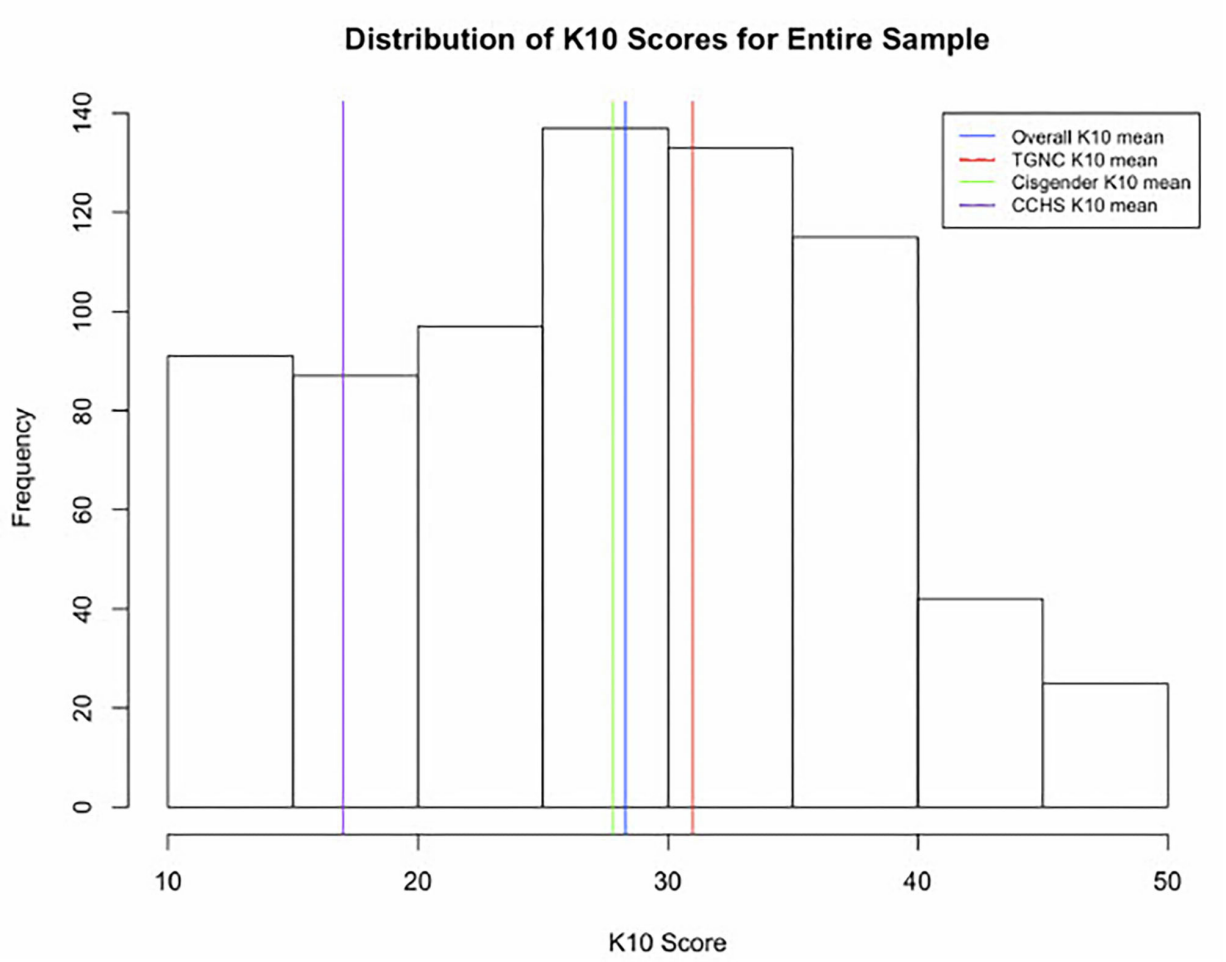
Distribution of K10 scores in the sample (*n* = 727). *The K10, or Kessler Psychological Distress Scale, is a standardized scale measuring psychological or mental health distress that was developed by Kessler (2002 and 2003). **For reference, K10 scores 10–15 “none to low physiological distress,” 16–21 “moderate psychological distress,” 21–29 “high psychological distress” and >30 “very high psychological distress.” ***CCHS, 2012 Canadian Community Health Survey-Mental Health data; ***TGNC, transgender and/or gender nonconforming.

The overall mean K10 score was 28 (IQR = 14.5, SD = 10), while the mean K10 for the TGNC group was 32 (IQR = 12, SD = 9) and the cisgender mean was 28.0 (IQR = 15, SD = 10). See Table 2 for descriptive results and Figure 1 for the distribution of K10 scores. Nearly half of the sample (48%, *n* = 346) fell within the highest range for the K10 cut points, with scores ranging from 30 to 50 indicating “very high distress.” Only 13% (*n* = 91) had scores from 10 to 15, which indicate “no to low psychological distress.” These cut points are taken from the 2007 Australian National Survey of Mental Health and Wellbeing (25).

**Table 2:** K10 scores by demographic variables.

**K10**		**mean** (***n***)
Entire sample		28.3 (727)
	IQR**	21.0, 35.5
	median	29.0
		**mean (** ***n*** **)**
TGNC*		31.3 (96)
	IQR**	26.0, 38.0
	median	32.5
Cisgender		27.8 (627)
	IQR**	20.0, 35.0
	median	28.0
**Sample in terms of K10 cut points**		***n*** **(%)**
10–15 none to low psychological distress		91 (13)
16–21 moderate psychological distress		103 (14)
22–29 high psychological distress		187 (26)
30–50 very high psychological distress		346 (48)
**K10 scores by Foundry Center**		**Mean (SD)**
	Vancouver Granville	28 (10.3)
	Kelowna	31 (9.2)
	Vancouver North Shore	27 (8.8)
	Prince George	28 (8.4)
	Victoria	30 (7.7)

* *TGNC = transgender and/or nonconforming*,

** *IQR = Interquartile range*.

### Results of Statistical Analyses

Results from the independent 2-sample *t*-test and age adjusted and unadjusted stratified linear regression models are explained here and presented in **Table 4**. The Cronbach's alpha for this sample was high, alpha = 0.93 (CI = 0.93, 0.94), demonstrating high item covariance, indicating that each item likely measures the same underlying concept. This alpha is the same or very similar to those found in the wider literature for the K10 (26–28). The results of the *t*-test indicated that, when compared with cisgender youth, TGNC youth showed significantly higher average scores on the K10 (difference in means = 3.44, *t* = 3.24, CI: 1.35, 5.52, and *p* = 0.001). Effect sizes for the overall difference between groups was medium (Cohen's *d*= 0.4*)*. The differences between groups in both the white and the non-white strata also showed a medium effect size (white strata, Cohen's *d* = 0.4 and non-white strata, Cohen's *d* = 0.4).

Both age unadjusted (see Table 3) and adjusted (see Table 4) models were trialed to explore the relationship between gender identity and mental health and the results from both models were nearly identical. Compared with cisgender youth, TGNC youth had significantly higher average K10 scores across both race strata (white strata: beta = 3.64, CI: 0.88, 6.40, *p* = 0.01 and non-white strata: beta = 3.42, CI: 0.02, 6.62, *p* = 0.04). Given the unadjusted and age adjusted models were almost identical, this suggested that age did not seem to substantially change the association between gender and mental health distress. This suggests that very little, if any, of the observed differences in mental health distress (as measured by the K10) between gender groups can be attributed to potential differences in age. Within the wider youth mental health literature, there is some support for the notion that age is associated with mental health distress, in that younger age might infer higher mental health distress but the results from this study suggested that age was not associated with the relationship of interest. Possible reasons for this are explored further in the Discussion section.

**Table 3:** Univariable analysis of gender identity and mental health distress, stratified by race.

	**Non-white strata**		**White strata**	
	Beta (95% CI***)	***p*** **-value**	Beta (95% CI***)	***p*** **-value**
Cisgender	REF**	–	REF**	–
TGNC*	3.42 (0.02, 6.63)	0.04	3.64 (0.88, 6.40)	0.01

* *TGNC = transgender and/or nonconforming*,

** *IQR = Interquartile range*.

**Table 4:** Multivariable analysis of gender identity, mental health distress and age, and stratified by race.

	**Non-white strata**		**White strata**	
	Beta (95% CI***)	***p*** **-value**	Beta (95% CI***)	***p*** **-value**
Cisgender	REF**	–	REF**	–
TGNC*	3.41 (0.20, 6.62)	0.04	3.60 (0.84, 6.37)	0.01
Age	−0.21 (−0.7, 0.3)	0.40	−0.06 (−0.5, 0.4)	0.80

* *TGNC, transgender and/or gender nonconforming*;

** *REF, referent group*;

*** *CI, confidence interval*.

## Discussion

The findings from the first objective show that TGNC young people scored significantly higher on a measure of mental health distress than their cisgender peers. This supports the original hypothesis that TGNC youth are more distressed than cisgender youth. Additionally, the results revealed the high mental health distress scores of the entire sample, as both TGNC and cisgender groups had scores in the high and very high categories on the K10 (overall mean = 28, TGNC mean = 31, cisgender mean = 28, see Table 3). This pattern was also seen across both race strata.

The results of this study are notable when compared to population level data from the 2012 Canadian Community Health Survey-Mental Health component (CCHS-MH) (29). First, the distress scores from this study, as measured by the K10, were considerably higher than population estimates for similarly aged youth, regardless of gender identity. Data from the 2012 CCHS-MH captured only binary options, male/female, and included K10 scores for respondents aged 15–24 years (*n* = 4,013), with a mean K10 score of 17 (SD = 9, median = 15). In comparing this estimate to the cisgender group in this study (i.e., comparing the two cisgender groups), the cisgender group in this study had higher average K10 scores (K10 = 28) than did the population group (K10 = 17), with an effect size of 1.2 (Cohen's *d* = 1.2). The study adds to the literature examining disparities in mental health among TGNC young people (30, 31).

High distress scores were expected in this sample given that it included young people who were accessing integrated health services at Foundry. The differences found represent a conservative estimate of the actual difference in mental health distress between TGNC and cisgender youth, as it is possible that the medium effect size seen between the cisgender and TGNC groups was obscured by the high rates of mental health distress among the cisgender group. If youth seeking services are overall more distressed than youth in the general population, and if TGNC youth are significantly more distressed than cisgender youth, it is important to examine the challenges and barriers TGNC youth might face in accessing effective and affirming care.

It is relevant to note that not all TGNC youth experience such high levels of mental health distress and it is likely that there are differences between TGNC population numbers and estimates gleaned from convenience samples. For example, findings from a US study revealed that TGNC young people accessing services from providers with some training on TGNC concepts and care, reported overall positive experiences with primary care (32). Results from a Canadian study of 839 TGNC youth reported that ~25% of the youth surveyed indicated their mental health was either good or excellent (33). However, in the absence of a large, population-based sample of TGNC youth in Canada, at this time it is not possible to comment on the extent of the mental health differences between TGNC youth in population vs. those seeking care. This points toward the need for a more comprehensive picture of the needs and wellbeing of TGNC young people in Canada, as these findings present only a small glimpse into one aspect of their mental health outcomes.

While these numbers illuminate the extent of between group differences, they do not explain *why* such differences might exist. What is important to emphasize is, it is *not* something inherent to TGNC identities that produce higher levels of mental health distress but rather, it is likely factors associated with social determinants (34–36). Research in the area of health outcomes for TGNC people has used the minority stress model (37, 38) to attempt to explain the health and mental health inequities seen in TGNC populations. Minority stress is defined as the process through which individuals belonging to multiple minority social groups experience stigma, prejudice and discrimination, resulting in an upsetting and stressful social experience (38). This model theorizes that the experience of discrimination and stigma from the social environment leads to health and mental health inequities for minority groups. Minority stress has been used to explain suicide risk in transgender adults in Canada (39), peer victimization, school belonging and drug use in trans youth and trans youth of color (40), depression and anxiety in TGNC youth (41), as well as used extensively to explain differences among sexual minority populations.

Regarding the second objective, the findings demonstrated that TGNC youth scored significantly higher than their cisgender peers, across both race strata. This suggests that support for the original hypothesis was not found and TGNC youth from this sample, at the intersection of TGNC identity and a non-white racial identity, do not appear to be significantly more distressed than white TGNC youth. There is limited research in this area, especially that takes an intersectional approach to mental health outcomes for TGNC youth and that examine race specifically. One study in the area pointed toward the importance of gender affirming health practices and also the need for cultural safety training in reducing the impact of both gender and racial discrimination for TGNC youth of color (4). Another study found that although transgender youth of color did experience higher rates of peer victimization (i.e., emotional, physical, or verbal abuse) than white transgender students, this did not lead to higher rates of drug use among transgender youth of color (despite victimization experiences leading to higher rates of drug use among white transgender students) (40).

To highlight the insights that can be revealed from an intersectional approach to a health research question, take another example from a 2014 study by Bostwick et al. This study adopted an intersectional approach to examine how suicide and self-injury factors among sexual minority youth (i.e., non-heterosexual identities and practices) differed according to sex and race/ethnicity (42). The results showed complex nuance within patterns of suicide and self-harm behaviors among sexual minority youth when stratified by race/ethnicity and then further, by sex. For example, Asian and Black sexual minority youth had better outcomes compared to white youth but when this was stratified further by sex, only Asian and Black female youth appeared to benefit from this protective factor (42). The authors of this study postulate the source of such protective factors to be within social and cultural conceptualizations of suicidality and mental health. This implies that the assumption that membership in *additional* minority groups infers poorer health outcomes is not necessarily supported by research, as can be seen other intersectional studies (4, 40, 42), and potentially in the results of this study. In terms of the results found for the study's second objective, it may be important for future research to consider the wide variety of experiences within a social category like non-white race, for example, looking at different subgroups like membership to a specific racial or cultural identity.

Collectively, this research and that of others (43) emphasizes the importance of adopting an intersectional approach to explicating health inequities for various populations, as traditional linear or additive methods may not fully “capture the intricate transactions between multiple social identities that shape the lived experiences” (35) of people as they navigate the social world. Assuming an intersectional approach may be beneficial to the design of the health system in general and integrated youth mental health services specifically because the interactions with health providers are sites of social engagement wherein stigma and discrimination are enacted (or vice versa, where respect and affirmation can occur). Research has shown intervenable factors to support TGNC persons and their mental health (39, 44, 45). This study contributes knowledge to the importance of person-centered care in health system design, taking into consideration the various needs of TGNC young people accessing integrated health services.

### Limitations

This study is not without limitations. WPATH recommendation for a two-step process includes questions about sex-assigned at birth and current gender identity that would allow for the possibility of a more nuanced understanding of gender identity that could include different combinations of sex and gender (5, 46). The survey used for this study did not follow this 2-part gender and sex item method and so this sample did not differentiate those individuals who reported male or female on the survey item and who also have a different birth-assigned sex, making the TGNC group potentially underrepresented. Similarly, the race variable was separated into white and non-white strata, thus limiting the potential to understand the nuance of reported distress across racial identities. Another limitation of this study is the cross-sectional nature of the quantitative data. This study represents a cross-sectional snapshot of mental health distress among TGNC youth and so no causation can be inferred. Future research in this area might consider a longitudinal approach to examining mental health distress and TGNC identity over time, that potentially includes qualitative narratives of how TGNC youth navigate service experiences as they move from adolescence to young adulthood and beyond.

Finally, a notable limitation of the study was the tool selected to measure distress. The K10 scale was originally designed as a population measure to capture non-specific psychological distress in an American adult population. Few studies have validated the K10 as a measure that is fit for purpose for the context of integrated youth health services. A recent, as yet unpublished, Canadian study that examined the validity of the K10 in the context of integrated youth health services (21), found that despite the K10's established statistical measurement performance, it lacks underlying conceptual validity within this context. This study points toward the need for future work to develop a conceptualization of mental health distress from the perspective of young people themselves, from which future measures can be validated and developed.

## Conclusion

This study found that TGNC young people were significantly more distressed than their cisgender peers at the point of data collection and that youth seeking care were significantly more distressed than youth in the population. It also found that while TGNC youth were more distressed than cisgender youth in both non-white and white race strata, there did not appear to be a specific, additional negative impact of a non-white racial identity on the mental health distress of TGNC young people. The application of intersectionality to this research contributed to a new conceptualization of health inequalities among TGNC young people and a recognition of the role multiple and intersecting pathways of oppression play in reproducing health inequalities. Further research and policies are needed that adopt this intersectional approach to better understand both the particular health outcomes and lived experiences of TGNC young people accessing health services, in a way that centers their concerns and supports those factors they define as contributing to their wellbeing. Given the context of this study, it is also recommended that existing provincial investments in integrated youth services, such as Foundry, are leveraged to support accessible, affirming and youth-friendly mental health care.

## Data Availability Statement

Data available on request due to privacy/ethical restrictions.

## Ethics Statement

This study involved human participants and was therefore were reviewed and approved by the University of British Columbia Behavioral Research Ethics Board. Written informed consent to participate in this study was provided by all participants.

## Author Contributions

RP, JP, AG, SM, and SB conceptualized study. RP wrote the manuscript. All authors contributed to the analysis, interpretation of results, and edited the final manuscript.

## Funding

This work was supported by the Canadian Institutes for Health Research (397474), University of British Columbia, Department of Psychiatry and the Canadian Occupational Therapy Foundation.

## Conflict of Interest

The authors declare that the research was conducted in the absence of any commercial or financial relationships that could be construed as a potential conflict of interest.

## Publisher's Note

All claims expressed in this article are solely those of the authors and do not necessarily represent those of their affiliated organizations, or those of the publisher, the editors and the reviewers. Any product that may be evaluated in this article, or claim that may be made by its manufacturer, is not guaranteed or endorsed by the publisher.

## Abbreviations

WHO: World Health Organization
TGNC: Transgender or gender non-conforming
WPATH: World Professional Association for Transgender Health
K10: Kessler Psychological Distress Scale
IQR: Interquartile range
SD: Standard deviation
CCHS-MH: Canadian Community Health Survey-Mental Health.
